# Hippocampal Hyperactivity as a Druggable Circuit-Level Origin of Aberrant Salience in Schizophrenia

**DOI:** 10.3389/fphar.2020.486811

**Published:** 2020-10-16

**Authors:** Dennis Kätzel, Amy R. Wolff, Alexei M. Bygrave, David M. Bannerman

**Affiliations:** ^1^Institute for Applied Physiology, Ulm University, Ulm, Germany; ^2^Department of Neuroscience, University of Minnesota, Minneapolis, MN, United States; ^3^Department of Neuroscience, Johns Hopkins University, Baltimore, MD, United States; ^4^Department of Experimental Psychology, University of Oxford, Oxford, United Kingdom

**Keywords:** schizophrenia, aberrant salience, glutamate hypothesis, attention, mesolimbic, hippocampus, CA3, subiculum

## Abstract

The development of current neuroleptics was largely aiming to decrease excessive dopaminergic signaling in the striatum. However, the notion that abnormal dopamine creates psychotic symptoms by causing an aberrant assignment of salience that drives maladaptive learning chronically during disease development suggests a therapeutic value of early interventions that correct salience-related neural processing. The mesolimbic dopaminergic output is modulated by several interconnected brain-wide circuits centrally involving the hippocampus and key relays like the ventral and associative striatum, ventral pallidum, amygdala, bed nucleus of the stria terminalis, nucleus reuniens, lateral and medial septum, prefrontal and cingulate cortex, among others. Unraveling the causal relationships between these circuits using modern neuroscience techniques holds promise for identifying novel cellular—and ultimately molecular—treatment targets for reducing transition to psychosis and symptoms of schizophrenia. Imaging studies in humans have implicated a hyperactivity of the hippocampus as a robust and early endophenotype in schizophrenia. Experiments in rodents, in turn, suggested that the activity of its output region—the ventral subiculum—may modulate dopamine release from ventral tegmental area (VTA) neurons in the ventral striatum. Even though these observations suggested a novel circuit-level target for anti-psychotic action, no therapy has yet been developed along this rationale. Recently evaluated treatment strategies—at least in part—target excess glutamatergic activity, e.g. N-acetyl-cysteine (NAC), levetiracetam, and mGluR2/3 modulators. We here review the evidence for the central implication of the hippocampus-VTA axis in schizophrenia-related pathology, discuss its symptom-related implications with a particular focus on aberrant assignment of salience, and evaluate some of its short-comings and prospects for drug discovery.

## What Does the Circuit-Level Origin of Schizophrenia Hold for Drug Discovery?

**Figure 1:**
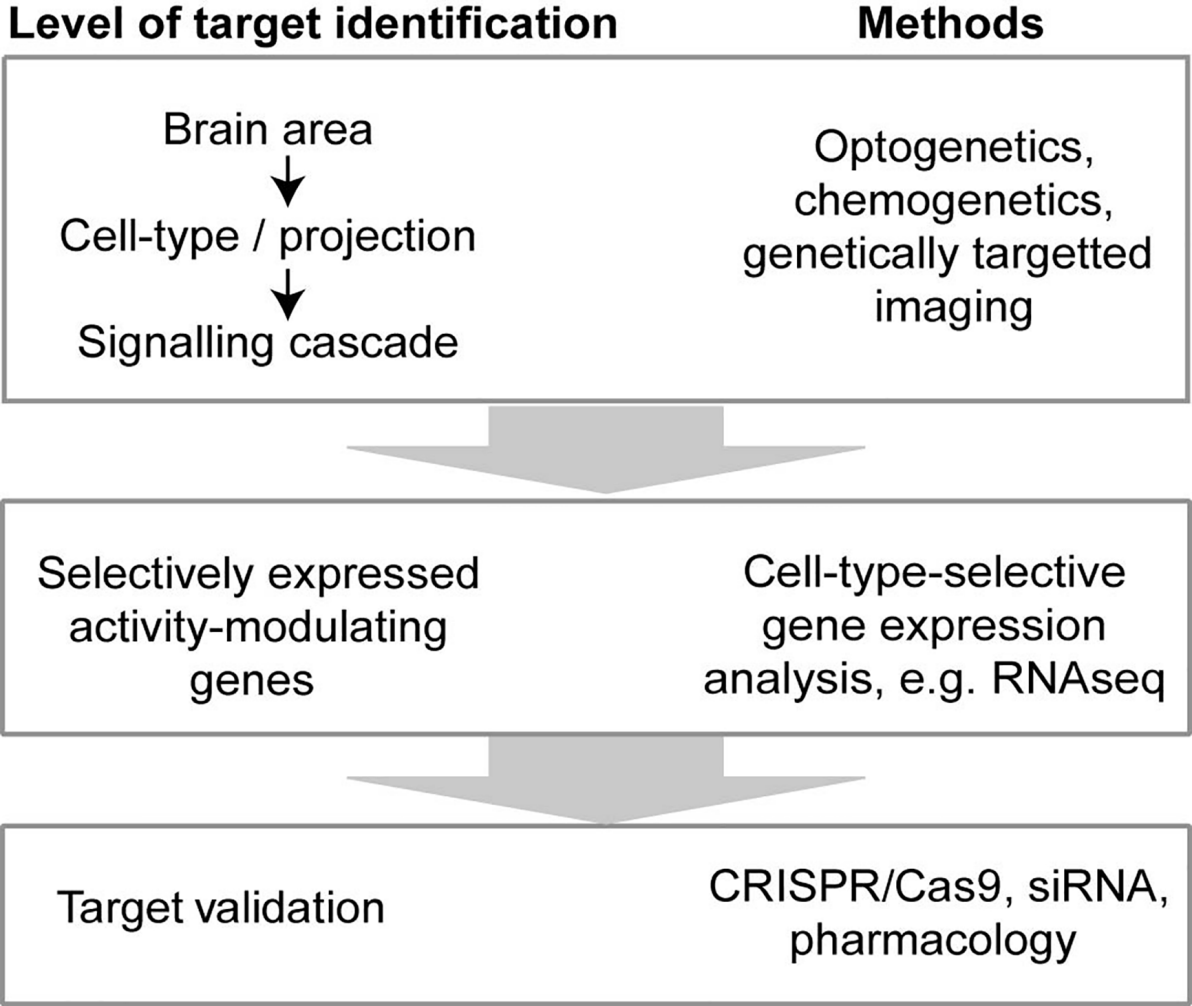
A circuit-neuroscience approach to drug discovery. Combining the optogenetic or chemogenetic modulation (Tye and Deisseroth, 2012; Deisseroth, 2014; Sternson and Roth, 2014) and imaging (Grewe et al., 2017; Ghosh et al., 2011) of genetically specified cell-types with behavioral testing in rodents, the brain areas, cell-types, specific projections, and potentially even signaling cascades that underlie certain cognitive functions can be identified (Kätzel and Kullmann, 2015). As a next step, genes selectively expressed in the identified cell-types can be revealed (Saunders et al., 2018; Tasic et al., 2018), which modulate neuronal activity. The proteins encoded by such genes can be ablated in these genetically specified cell-types using e.g. CRISPR/Cas9 (Swiech et al., 2015), or modulated systemically by pharmacology to validate their suitability as molecular treatment targets.

The principle need for drug discovery in schizophrenia (SCZ) arises from the considerable extent of treatment-resistance in this disease; negative and cognitive symptoms respond only poorly, if at all, to currently available anti-dopaminergic neuroleptics and even positive symptoms remain refractory—even to clozapine, the only approved drug for pharmacoresistant schizophrenia—in ca. 30 % of patients (Owen et al., 2016; MacKay et al., 2018). The principal value-proposition that modern circuit neuroscience holds for psychiatric drug discovery is that it allows the identification of genetically-specified *cellular* targets for treatment, and enables their translation into *molecular* targets (see **Figure 1**).

By this generic strategy, individual cell-types in specific brain regions are modulated by opto- or chemogenetics in awake rodents, while schizophrenia-related behavioral or physiological parameters are recorded. In this way, individual cell-types and, subsequently, molecular signaling cascades within them are singled out as putative cellular treatment targets. For example, two schizophrenia-related landmark studies, showed that the activation of the G_q_-protein cascade through the synthetic receptor hM3Dq specifically in parvalbumin-positive (PV) interneurons of the dorsal CA1-region of the hippocampus could improve schizophrenia-related physiological and behavioral deficits in a mouse model carrying a human genetic risk factor of schizophrenia (*Lgdel*-hemideletion) (Marissal et al., 2018; Mukherjee et al., 2019). This singles out G_q_-coupled receptors in hippocampal PV interneurons as putative drug targets in schizophrenia. As a next step, large-scale datasets from single-cell genome-wide gene expression studies can be harnessed (Saunders et al., 2018; Hodge et al., 2019) to assess which receptors (e.g. GPCRs, in this example) or other activity-modulating proteins are expressed in this cell-type with relative specificity, thereby converting the identified cellular target into a molecular target. Finally, ablating such target genes specifically in those cell-types or manipulating them pharmacologically, if possible, during the same *in vivo* tests, can validate their suitability as targets pre-clinically (**Figure 1**).

Given these opportunities, a “circuit view” of relevant psychological functions promises to uncover a wider range of potentially very specific novel targets; e.g. in the form of genes selectively expressed in only a specific subset of neurons in those circuits in which the pathological development of schizophrenia takes place. This likely exceeds the identifiable pharmacological options that a “synaptic view”—such as focussing on the key players of a “generic” glutamatergic or dopaminergic synapse—would provide.

## Dopaminergic Pathologies and Aberrant Salience as the Final Common Pathway in Schizophrenia

All currently used neuroleptics have in common that they act as antagonists of D2-type dopamine receptors (D2Rs). Hence, when searching for the circuit-level origin of schizophrenia, dopaminergic aberrations are a promising place to start. As reviewed in (Howes et al., 2015), patients with schizophrenia show increased striatal presynaptic dopamine synthesis capacity (and availability), increased induced dopamine release (e.g. after amphetamine), and mildly increased striatal D2/3-receptor density. A key question, however, is how this hyper-dopaminergic phenotype relates to positive (and potentially other) symptoms of schizophrenia.

One candidate as a psychological cause for hallucinations and delusions in schizophrenia is the malfunctioning of the mechanisms in the brain that assign salience, importance and meaning to items of perception and thought (Gray et al., 1991). This framework was originally developed by Manfred Spitzer (1995), who argued: “… the question is no longer, ‘How does somebody arrive at a false statement?’. Instead, we must ask, ‘How does the process of assigning significance to things or events become disturbed?’, and ‘How do the capabilities for learning and change become disturbed?’” (Spitzer, 1995). Referring to the ability of amphetamine to produce psychotic symptoms, he argued that aberrant dopamine and/or noradrenaline signaling could underlie the erroneous attribution of significance that leads to the formation of delusions (Spitzer, 1995). He further suggested that the combination of reduced *tonic* dopamine release in the nucleus accumbens (NAc), the adaptive upregulation of NAc dopamine receptors, and resulting hypersensitivity to *phasic* dopamine release (Grace, 1991) could represent a mechanism underpinning this process (Spitzer, 1995).

Shitij Kapur (Kapur, 2003) developed these ideas further, emphasizing that, physiologically, the activity of dopamine neurons in the ventral tegmental area (VTA) is thought to serve as a reinforcing teaching signal encoding the error made by the brain’s internal prediction process (Ljungberg et al., 1992; Montague et al., 1996; Schultz et al., 1997; Schultz, 2007). His conclusion was that erroneous prediction signals, likely corresponding to inappropriately increased phasic activity of mesolimbic VTA dopamine neurons, underlies the formation of false perceptions and beliefs, and hence hallucinations and delusions in SCZ (Kapur, 2003; Winton-Brown et al., 2014). Fittingly, activity in the mesolimbic target region, the NAc, encodes prediction errors, associated with rewarding stimuli (Apicella et al., 1992; Schultz et al., 1992; Heinz and Schlagenhauf, 2010). Beyond that, mesolimbic, nigrostriatal, and mesocortical dopamine also encode the salience of stimuli and arousal signals unrelated to reward (Horvitz, 2000; Heinz and Schlagenhauf, 2010; Boehme et al., 2015).

The implication of this “aberrant salience” theory of psychosis is that delusions are the result of the brain’s attempt to make sense of a neural representation of a world in which items have been assigned inappropriately high significance. Inferring “sense” from a representation of the world which renders unimportant stimuli as highly salient seems an unsolvable mental task, and delusions may be the inevitable consequence of such attempts (Spitzer, 1995; Kapur, 2003), as Kapur (2003) has described in greater detail. In his view, the concrete contents of delusions represent a somewhat coherent explanation for the unusually high and persistent significance that items of perception and thought get assigned by the patient’s brain (involving striatal dopamine release). Hallucinations, in turn, could be a more direct consequence of the unusually high salience that the internal representations of imagery, sensory percepts (or their memories) and thought processes get assigned (Kapur, 2003).

Using psychological tests of salience attribution, this hypothesis has received empirical support. Patients with schizophrenia display higher aberrant salience in terms of falsely assigning predictive value to non-predictive cues (Katthagen et al., 2016). Importantly, the error rate resulting from the assignment of inappropriately high predictive power to non-predictive cues correlates with the severity of positive symptoms (Morris et al., 2013). Also, patients that still experience delusions despite medication show higher aberrant salience attribution than treatment-responsive patients do (Roiser et al., 2009). Further, prodromal patients at *ultra-high risk* (UHR) of developing overt schizophrenia also showed increased aberrant salience (Roiser et al., 2013), arguing for a causal relationship between an (earlier) abnormality of salience attribution and (resulting) psychosis.

## Aberrant Physiological Patterns of Salience Representation in Schizophrenia

Several combined behavior/functional magnetic resonance-imaging (fMRI) studies support the notion of a link between schizophrenia, aberrant attribution of salience to sensory stimuli, and altered striatal dopamine signaling (Heinz and Schlagenhauf, 2010; Roiser et al., 2013; Winton-Brown et al., 2014). For example, larger ventral striatal dopamine synthesis capacity is associated with increased aberrant salience in healthy humans (Boehme et al., 2015), and abnormal reward prediction signals have been found in the ventral striatum of schizophrenia patients (Juckel et al., 2006). The consequences are described tellingly by Kapur (2003): “Dopamine *mediates* the process of salience acquisition and expression, but under normal circumstances it *does not create* this process. It is proposed that in psychosis there is a dysregulated dopamine transmission that leads to stimulus-independent release of dopamine. This neurochemical aberration usurps the normal process of contextually driven salience attribution and leads to *aberrant assignment of salience to external objects and internal representations*. Thus, dopamine, which under normal conditions is a mediator of contextually relevant saliences, in the psychotic state becomes a creator of saliences, albeit aberrant ones.” The observation that *acute* intake of amphetamine does not cause psychotic symptoms in healthy humans (Yui et al., 1999), but is sufficient in stabilized schizophrenia patients to re-instantiate their positive symptoms (Angrist et al., 1974; Yui et al., 1999) appears to be key to this notion (Kapur, 2003): it is not that an acute excess of dopamine simply translates into excess salience attribution, but it is only detrimental once salience-related circuitry is already altered, which *might* or *might not* itself be caused by chronically increased dopaminergic signaling in schizophrenia.

These observations point beyond a simplistic model of an *ad-hoc* increase of salience assignment to irrelevant stimuli by pathologically high striatal dopaminergic signaling in established schizophrenia. Instead, the core problem is that aberrant mesolimbic dopamine—since it is a teaching signal—drives maladaptive associative learning over years as the disease develops. Both, Kapur (2003) and Spitzer (1995) provide this emphasis on the associative and, hence, predictive nature of salience attribution. On the one hand, this relates to *theoretical frameworks* that view aberrations in the brain’s ongoing process of making predictions about imminent sensory experiences as central to schizophrenia (Fletcher and Frith, 2009; Rentzsch et al., 2015). On the other hand, this notion links to the pathological emergence—caused by maladaptive learning or impaired brain development, or both—of the neural mechanisms that govern such associations, over a longer time-scale (Kapur, 2003). This is evidenced by the documentation of the psychological changes that prodromal patients experience over long periods before their first psychosis (Kapur, 2003) and the profound chronic alterations of salience-related physiological response patterns in unmedicated first-episode patients (Knolle et al., 2018). The latter was studied in healthy controls and unmedicated first-episode SCZ-patients by combining fMRI with a visual oddball-paradigm displaying images that would fall into different categories of salience (novelty, negative emotion, task-driven salience). Group-differences in the activation of various brain regions emerged depending on the type of salience. In all cases, the relative cue-induced activity change of the respective region was *opposite* in SCZ patients (reduction) compared to controls (increase)—which included changes in the dopaminergic midbrain (VTA, SNc: group-difference in *all* types of salience), the amygdala, anterior cingulate cortex, and parahippocampal gyrus (difference for negative emotional salience), as well as the striatum and cerebellum (difference for novelty and negative emotional salience) (Knolle et al., 2018). However, within the group of patients, SNc/VTA-activation by novelty was strongly positively correlated with both hallucinations and negative symptoms. Similarly, the activation of the striatum and amygdala by emotional salience correlated with positive symptoms (Knolle et al., 2018).

A study in ultra-high-risk (UHR) patients found that delusion-like symptoms are correlated positively with aberrant reward prediction error signals in the ventral striatum, supporting a mechanistic link between aberrant salience-related mesolimbic signaling and delusions (Roiser et al., 2013). However, this study also suggested that the dynamics of physiological signals that occur in response to salient cues changes *altogether* in UHR-patients: a positive correlation between ventral striatal responses to distractive cues and the extent of aberrant salience attribution seen in control subjects, was not apparent in UHR individuals (Roiser et al., 2013). Thus, the physiological operation (or “structure”) of salience attribution is fundamentally altered in this disease early on, as proposed by Spitzer by using the term “deformed structure” (Spitzer, 1995).

Hence, the limited efficacy of anti-dopaminergic treatment in established schizophrenia may be related to the fact that it is neither fine-grained enough to correct pathological salience attribution processes nor can it rewind the psychological result [“deformed structure” (Spitzer, 1995)] of years of maladaptive learning processes caused by it. Kapur (Kapur, 2003) pointed out, that D2-related antipsychotics often do not lead to an abolishment of hallucinations and delusions, but to a decrease of their perceived significance for the patient (Miller, 1989; Chouinard and Miller, 1999). This implies that anti-dopaminergic neuroleptics may not alleviate the upstream cause of the dopaminergic dysregulation, but rather tune out some of its downstream consequences (Kapur and Remington, 2001).

These observations put particular emphasis on *early interventions*, preferably in the prodromal state, for future therapies. These therapies would need to counter the maladaptive learning by normalizing aberrant dopamine signaling. While broad anti-dopaminergic treatment is likely not an option given its non-specific nature and resulting unwanted side-effects like excessive reduction of selective attention, motivation or movement, a modulation of the neural circuits that *control* the dopaminergic system – or even non-dopaminergic mechanisms of salience assignment (Knolle et al., 2018) - could provide opportunity for more tailored interventions. Historically, these efforts have focused on understanding glutamatergic circuit pathologies upstream of dopaminergic dysregulation (Lisman et al., 2008; Coyle et al., 2010; Coyle, 2012; Moghaddam and Krystal, 2012; Barkus et al., 2014).

## Upstream Circuits Regulating Dopaminergic Activity: Hippocampal Hyperactivity in Patients

In search of the brain structures that might be causing aberrant dopaminergic activity, actual physiological endophenotypes seen in patients may provide helpful guidance (Kellendonk et al., 2009; Bolkan et al., 2015). Among the most robust and replicated physiological endophenotypes is a pathological hyperactivity of the anterior hippocampus and surrounding cortical regions (Heckers and Konradi, 2015) both at rest (Medoff et al., 2001; Schobel et al., 2009; Schobel et al., 2013; Talati et al., 2014; Tregellas et al., 2014; Talati et al., 2015; Talati et al., 2016) and during minimal cognitive engagement by sensory stimulation or demands for visual fixation (Malaspina et al., 1999; Holt et al., 2005; Holt et al., 2006; Tregellas et al., 2009). This basal hyperactivity is already present in early stages of the disease (McHugo et al., 2019). A landmark longitudinal imaging study in prodromal (UHR) patients demonstrated that the only neurophysiological aberration that could—with any likelihood—predict transition to psychosis was elevated cerebral blood volume (CBV) in the anterior CA1 region (Schobel et al., 2009; Schobel et al., 2013). It was also shown that transition to overt schizophrenia (characterized by psychosis) was accompanied by elevated CBV in the anterior subiculum and atrophy of the anterior hippocampus (Schobel et al., 2013), which also affects the posterior hippocampus later (McHugo et al., 2018), and spreads to all hippocampal subfields after starting in CA1 (Ho et al., 2017). Therefore, this biomarker of anterior hippocampal hyperactivity has considerable significance in potentially leading the way toward early interventions that could prevent disease progression, spreading hippocampal atrophy, and psychosis (Insel et al., 2010; Moghaddam, 2013; Schobel et al., 2013; McHugo et al., 2018). Notably, physiological hyperactivity of the hippocampus, as determined by cerebral blood volume (CBV), is correlated with the severity of positive and cognitive symptoms (Schobel et al., 2009; Schobel et al., 2013; Tregellas et al., 2014). For example, there is a negative correlation between hippocampal hyperactivity and working memory performance in schizophrenia patients (Tregellas et al., 2014). This baseline hyperactivity may also lead to a reduction in any task-related activation of the anterior hippocampus, as shown during a memory task and visual stimulation in schizophrenia patients (Heckers et al., 1998; McHugo et al., 2019).

Seminal fMRI studies from the laboratory of Stephan Heckers linked hippocampal hyperactivity directly to pathological salience attribution, demonstrating a failure in patients to reduce the physiological representation of the salience of a stimulus to its decreasing novelty-related relevance [akin to short-term habituation (Holt et al., 2005; Barkus et al., 2014)]. The researchers presented intrinsically salient stimuli (fearful faces) to patients with schizophrenia and control subjects, and found that the hippocampus showed increased activity in response to these stimuli. However, while the hippocampal BOLD-signal evoked by the stimulus decreased with repeated stimulus presentations in healthy controls, it remained relatively constant or even slightly increased in schizophrenia patients (Holt et al., 2005). In two follow-up studies, Heckers and colleagues demonstrated that the physiological habituation-deficit was also apparent when using neutral stimuli, and that it is already present in early-stage schizophrenia (Williams et al., 2013; Avery et al., 2019).

## The Ventral CA1/Subiculum→NAc→VTA Circuit and Control of Dopaminergic Activity in Rodents

**Figure 2:**
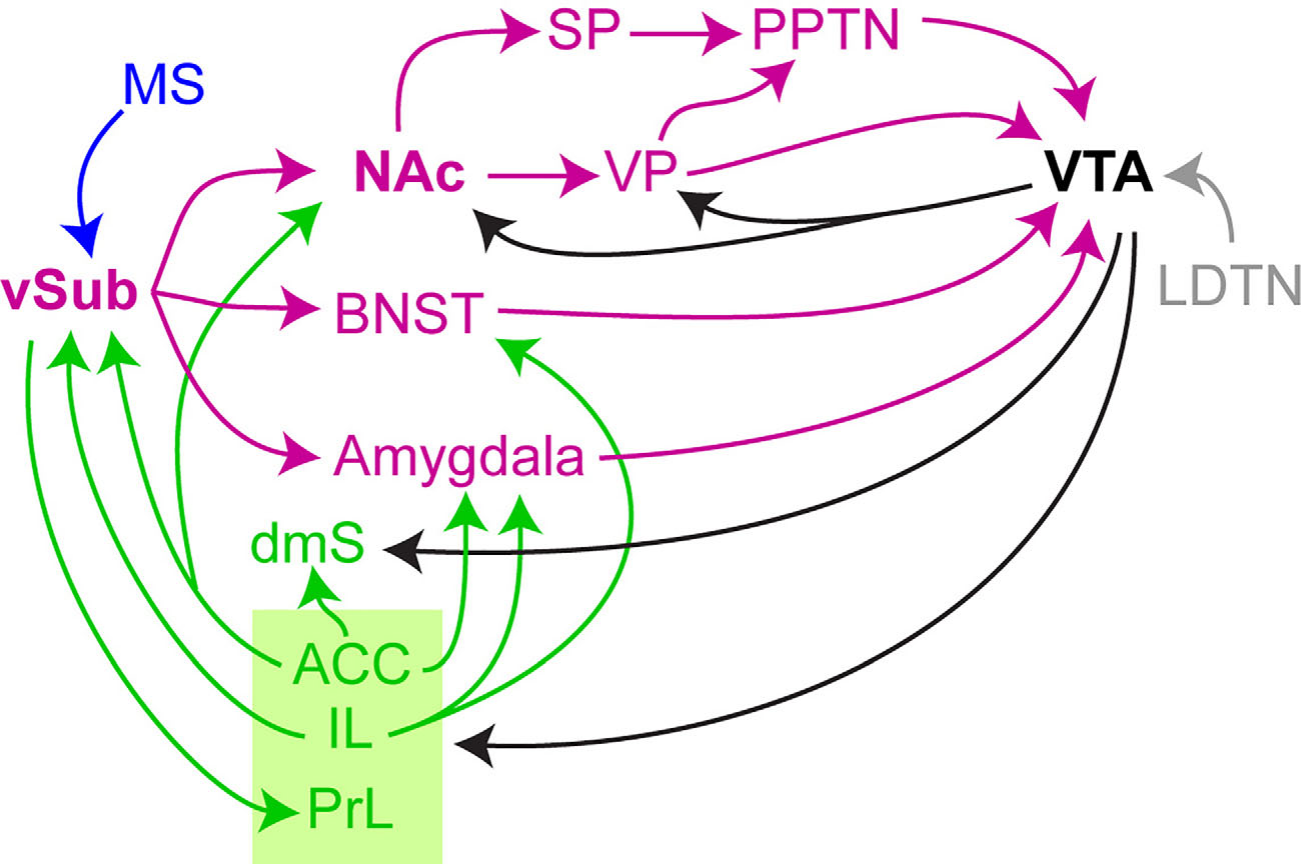
Ventral hippocampal projections that regulate dopamine neuron activity and release. Different, but interacting circuits have been found to regulate dopaminergic signaling by the ventral tegmental area (*VTA*) (see main text and **Tables 1**-**3** for description and references): projections from the ventral subiculum (*vSub*, the output region of the vHC) target the VTA through the bed nucleus of the stria terminalis (*BNST*), the amygdala or through the ventral basal ganglia comprising the nucleus accumbens (*NAc*) and the ventral pallidum (*VP*) thereby determining the number of active dopamine neurons. Additional input from the pedunculo-pontine tegmental nucleus (*PPTN*) and the latero-dorsal tegmental nucleus (*LDTN*) determine if these active neurons enter into burst-firing mode with ensuing phasic dopamine release in the NAc. The PPTN is modulated indirectly by the vSub→NAc pathway *via* neurons in the subpallidal region (*SP*), comprising part of the VP, the lateral hypothalamus, and the substantia innominata. Medial septal (*MS*, blue) and prefrontal influences (green) from the infralimbic (*IL*) and anterior cingulate (*ACC*) cortex onto dopaminergic neurons act centrally through the vSub as well, but also through the NAc, BNST, and amygdala; the ACC also innervates the dorsomedial (associative) striatum (*dmS*) which has gained increasing attention as a key target of dopaminergic midbrain projections aside from the ventral striatum (NAc).

While a mechanistic causal relationship between hippocampal hyperactivity and dopaminergic dysregulation remains to be established in humans, the rodent literature provides ample evidence for this link. In rodents, the hippocampus and some of its direct and indirect projection targets have been associated with the control of dopaminergic signaling, as summarized in **Figure 2** and **Table 1**.

**Table 1:** Experiments demonstrating the control of mesolimbic dopamine activity by the rodent ventral hippocampus and associated circuits.

Manipulation	Structure	Consequence	Reference
Electrical, 20 Hz	vSub	sustained DA release in the ipsilateral NAc which is dependent upon glutamatergic activity in both VTA & NAc	(Blaha et al., 1997; Taepavarapruk et al., 2000; Howland et al., 2004; Taepavarapruk et al., 2008)
Chemical stim.*	vSub/vHC	activates VTA DA neurons and DA release in NAc, VTA & PFC	(Legault and Wise, 1999; Legault et al., 2000; Mitchell et al., 2000; Floresco et al., 2001; Lodge and Grace, 2005; Peleg-Raibstein et al., 2005)
Electrical	vSub/vHC	activates NAc neurons that project to VP which projects to the VTA vSub-presynapses in the NAc are themselves regulated by VTA activity *via* DA release in the NAc and D1Rs/D2Rs vHC-stimulation increases the *number* of spontaneously active VTA neurons through the VP→VTA pathway while concomitant activation of afferents from the PPTN into the VTA increases *burst firing*, albeit exclusively in the already spontaneously active VTA neurons (determined by the vSub), thereby elevating DA release in the NAc the PPTN is modulated by D2Rs and input from a vHC-NAc-subpallidum pathway LDTN→VTA afferents are required for burst-firing of DA neurons	(Yang and Mogenson, 1984; Yang and Mogenson, 1985; Floresco et al., 2001) (Yang and Mogenson, 1986; Blaha et al., 1997; Floresco et al., 2001) (Lodge and Grace, 2005; Grace, 2010) (Yang and Mogenson, 1987) (Lodge and Grace, 2006)
Electrical	vSub/vHC	increases VTA DA activity through glutamatergic activation of the *bed nucleus of the stria terminalis* (BNST), which provides glutamatergic and GABAergic inputs to the VTA, mostly modulating DA neurons *via* VTA interneurons sustained DA hyperactivity caused through NMDAR-dependent long-term plasticity of the vSub-BNST projection	(Georges and Aston-Jones, 2002; Jalabert et al., 2009; Kudo et al., 2012; Jennings et al., 2013; Glangetas et al., 2015) (Glangetas et al., 2015)
Electrical	vSub	increases DA release in PFC, relying on GluRs in VTA & PFC	(Taepavarapruk et al., 2008)
Chemical inhibition (TTX)	IL	increases VTA-DA neuron activity *via* an increase of vSub-activity *Note:* PFC activity is also *necessary* to enable the activation of NAc-cells by the vSub, unless the vSub-NAc pathway has been potentiated; this vSub-NAc LTP can be reversed by PFC-inactivation *if* D2Rs are blocked	(Belujon and Grace, 2008; Patton et al., 2013)
Chemical stim.*	IL	decreases VTA-dopamine neuron activity *via* activation of the BLA	(Patton et al., 2013)
Electrical	IL	Increases BNST activity and thereby VTA activity	(Massi et al., 2008; Jalabert et al., 2009)
Chemical stim.	MS	increases VTA DA neuron activity and decreases SNc DA neuron activity through its action on the vSub	(Bortz and Grace, 2018a; Bortz and Grace, 2018b)
MAM-model	vHC	hyperactivity of the vSub & increased number of spontaneously active VTA DA neurons which can be normalized by pharmacological inhibition of the vHC	(Lodge and Grace, 2007; Lodge and Grace, 2008)
Cyclin-D2-KO	vHC	Hyperactivity throughout the vHC & increased number of spontaneously active VTA DA neurons which can be normalized by implantation of GABAergic precursor cells into the vHC	(Gilani et al., 2014)
Chemical inhibition (TTX)	vHC	prevents increase of DA release in ipsilateral NAc evoked by spatial novelty (but without decreasing exploratory activity)	(Legault and Wise, 2001)
GluR-inhibition	VTA	prevents increase of DA release in ipsilateral NAc evoked by spatial novelty (but without decreasing exploratory activity)	(Legault and Wise, 2001)

MAM, methylazoxymethanol acetate applied prenatally - developmental rat model of schizophrenia; BLA, basolateral amygdala; BNST, bed nucleus of the stria terminalis; DA, dopamine; Electrical, electrical stimulation (in most cases at 20 Hz); GluR, ionotropic glutamate receptors; IL, infralimbic prefrontal cortex; NAc, nucleus accumbens; LDTN, latero-dorsal tegmental nucleus; PFC, prefrontal cortex; PPTN, pedunculo-pontine tegmental nucleus; stim., stimulation; vHC, ventral hippocampus; VP, ventral pallidum; vSub, ventral subiculum (the main output region of the vHC); VTA, ventral tegmental areal; *chemical activation through infusion of NMDA or bicuculline.

It has repeatedly been demonstrated that electrical or chemical stimulation of the subicular output region of the rodent homolog of the anterior hippocampus—namely the ventral hippocampus (vHC)—increases the number of active dopaminergic neurons in the VTA and provokes dopamine release in the nucleus accumbens (NAc), which is considered to underlie the emergence of positive symptoms of schizophrenia ((Lisman et al., 2008; Grace, 2012; Perez and Lodge, 2014); see **Table 1** for a detailed list of studies and findings). Anatomically, however, this causal influence is rather indirect and can involve multiple routes either through the basal ganglia (Nucleus accumbens, NAc, and ventral pallidum, VP, the bed nucleus of the stria terminalis, BNST, or the amygdala **Figure 2**.

These projections are further embedded in a wider “circuit of circuits”, including the prefrontal (PFC) and anterior cingulate (ACC) cortex which—alongside the subdivisions of the striatum—rank among the most prominent output targets of the dopaminergic midbrain (**Figure 2**) (Taepavarapruk et al., 2008; Patton et al., 2013; Glangetas et al., 2015; Decot et al., 2017). Multiple uni- and bidirectional connections between these structures and selective targeting of interneurons within these circuits, complicate the situation considerably, as for example illustrated by the dopaminergic control of the vHC-NAc projection (**Table 1**).

Notably, dopaminergic projections to the associative striatum have also been implicated. For example, higher D2R-availability was found in schizophrenia only in the *dorso-medial* (associative)—but not the ventral—striatum (Kegeles et al., 2010), and the same regional specificity holds for dopamine synthesis capacity (^18^F-DOPA uptake) in prodromal patients (Howes et al., 2009). However, it has been suggested that the mesolimbic VTA→NAc pathway in rodents is partly homologous to the projection from the dopaminergic midbrain to the associative striatum in humans (Nauta et al., 1978; Ikeda et al., 2013; McCutcheon et al., 2019), implying that some of the rodent findings outlined in **Tables 1****–****3** could correspond to alterations of the dopaminergic innervation of the associative striatum in human schizophrenia. Hence, the exact and potentially distinct nature of schizophrenia-related dopaminergic dysfunction in these two subregions of the striatum and their corresponding dopaminergic input streams (VTA, SNc) remains to be fully resolved (Weinstein et al., 2017).

**Table 2:** Experiments demonstrating the dependence of hyperlocomotion, pre-pulse inhibition (PPI), and salience attribution on striatal dopamine.

Manipulation	Structure	Consequence	Ref
Chemogenetic activation	VTA; VTA→NAc	Provokes sustained *hyperlocomotion*; (*not* caused by activation of the SNc instead of VTA)	(Boekhoudt et al., 2016)
DA↓ *	VTA, SNc	blunts amphetamine-induced *hyperlocomotion*; restored by selective rescue of DA release in the NAc	(Heusner et al., 2003)
DA↑**/**D2R ↑ **	NAc, mSt	disrupts *PPI*	(Swerdlow et al., 1990b; Swerdlow et al., 1992; Wan and Swerdlow, 1993; Wan et al., 1994)
NMDAR↓ ***	VTA, SNc	reduces burst-firing of DA neurons; impairs various forms of associative learning, especially leading to erroneous *generalization* during associative fear-learning, potentially reflecting impaired selective salience assignment	(Zweifel et al., 2009; Parker et al., 2010; Zweifel et al., 2011)

D2R, dopamine receptor type 2; DA, dopamine; mSt, medial striatum; NAc, nucleus accumbens; NMDAR, NMDA-type glutamate receptor; SNc, pars compacta of the substantia nigra—referring to direct manipulation of DA neurons in the context of this table; VTA, ventral tegmental areal—referring to direct manipulation of DA neurons in the context of this table; *cell-type-selective tyrosine-hydroxylase ablation; **intra-accumbal infusion of D2R agonist, DA, or amphetamine; ***genetic ablation of NMDA-receptors from dopamine transporter (DAT) positive neurons (majority of DA neurons in SNc and VTA);

**Table 3:** Experiments demonstrating the control of dopamine- and salience-related behaviors by the rodent ventral hippocampus.

Manipulation	Structure	Consequence	Ref
Electrical (20** Hz**)	vSub	provokes *hyperlocomotion;* normalized by systemic D1R-antagonism or local blockade of NAc AMPARs, but not by systemic D2R-antagonism (raclopride)	(Taepavarapruk et al., 2000)
Optogenetic stim. (20 Hz)	vSub	provokes *hyperlocomotion;* limited responsiveness to D2R-antagonism (raclopride)	(Wolff et al., 2018)
Chemical stim.*	vHC	provokes *hyperlocomotion;* reduced by D2-*agonist* administration into the NAc and by systemic haloperidol and clozapine	(Yang and Mogenson, 1987; Legault and Wise, 1999; Bast et al., 2001a; Wolff et al., 2018)
Chemical disinhibition**	vHC	provokes *hyperlocomotion* and may disrupt *PPI* in dependence on the strain	(Bast et al., 2001b; McGarrity et al., 2017)
Chemogenetic disinhibition	vHC	provokes *hyperlocomotion* and disrupts *PPI*	(Nguyen et al., 2014)
Chemical inhibition***	vHC	decreases novelty-induced locomotor activity	(Bast et al., 2001c)
Electrical (20** Hz**)	vSub	disrupts *PPI*	(Howland et al., 2004)
Chemical stim.*	vHC	disrupts *PPI;* not normalized by haloperidol or clozapine	(Bast et al., 2001a)
Optogenetic stim. (20 Hz)	vSub	impairs *spatial novelty-preference*	(Wolff et al., 2018)
Chemical disinhibition**	vHC	decreases *attentional accuracy* in the 5-CSRTT increases *inattentiveness* (omissions) in the 5-CSRTT	(McGarrity et al., 2017) (Tan et al., 2018)
Optogenetic disinhibition****	vHC	increases *inattentiveness* (omissions) in the 5-CSRTT	(Tan et al., 2018)
MAM-rat model (increased vSub & VTA activity)	–	enhanced *hyperlocomotion* in response to PCP and amphetamine; however the sole dependence of this deficit on vHC-alterations remains to be determined	(Lodge and Grace, 2009; Gastambide et al., 2012)
CD2-KO mouse (increased vHC & VTA activity)	–	*novelty-induced hyperlocomotion*, normalized by mGluR2/3-agonist but not by selective D2R- or D1R-antagonists	(Gilani et al., 2014; Grimm et al., 2018)

Studies documenting aberrations in dopamine- and salience-related behaviors after experimental manipulations that cause hyperactivity of the ventral hippocampus. We also include measures of attention in the 5-CSRTT as they are dependent on dopaminergic signaling in the NAc (Pezze et al., 2006) and may reflect salience attribution to the attended visual cues. 5-CSRTT, 5-choice-serial-reaction time task; AMPAR, AMPA-type of glutamate receptor; CD2-KO, cyclin-D2 knockout mouse; Electrical, electrical stimulation (in most cases at 20 Hz); MAM, methylazoxymethanol acetate applied prenatally - developmental rat model of schizophrenia; NAc, nucleus accumbens; PCP, phencyclidine (an NMDA-receptor blocker used to model aspects of schizophrenia); PPI, pre-pulse inhibition; vHC, ventral hippocampus; vSub, ventral subiculum (the main output region of the vHC); VTA, ventral tegmental areal; *NMDA-infusion, **picrotoxin infusion, ***TTX infusion, ****silencing of vHC interneurons expressing the schizophrenia risk gene ErbB4.

## The Ventral CA1/subiculum→NAc→VTA Circuit and Control of Dopamine-Related Behavioral Readouts in Rodents

Several behavioral phenotypes have been linked to increased VTA-activity and ensuing striatal dopamine release (**Table 2**). This includes an augmentation of *locomotor hyperactivity* (hyperlocomotion) induced by spatial novelty or psychostimulants. Amphetamine-induced hyperlocomotion is considered to be a putative rodent correlate of positive symptoms of schizophrenia (Arguello and Gogos, 2006), based on the underlying causation of a hyperdopaminergic state by amphetamine and on its responsiveness to antipsychotics (Arguello and Gogos, 2006; Wilson and Terry, 2010). The augmentation of novelty-induced hyperlocomotion, which is also responsive to anti-dopaminergic treatment (Grimm et al., 2018), in turn, may relate to a failure to reduce novelty-related salience attribution to spatial stimuli as they become familiar (Barkus et al., 2014). A further dopamine-related readout that can also be observed as an endophenotype in patients with schizophrenia is reduced *pre-pulse inhibition* (PPI) (Geyer and Swerdlow, 1998; Braff et al., 2001; Ludewig et al., 2002; Swerdlow et al., 2008; Swerdlow et al., 2016). PPI is a pre-attentional form of the stimulus-specific regulation of reactivity to highly salient sensory stimuli, also termed sensorimotor gating. It is impaired by systemic amphetamine (Geyer et al., 2001) and local D2-specific dopaminergic agonism in the NAc (Swerdlow et al., 1990a; Swerdlow et al., 1992; Wan and Swerdlow, 1993; Wan et al., 1994) through a projection from the NAc to the ventral pallidum [VP; (Swerdlow et al., 1990b)] to the peduncolo-pontine tegmental nucleus [PPTN; (Swerdlow et al., 2016)] making it a valuable readout of maladaptive dopaminergic regulation of this circuit.

Importantly, artificially increasing ventral hippocampal output by stimulation of the vHC or vSub likewise reliably causes behavioral phenotypes that are typically associated with elevated dopaminergic signaling, including locomotor hyperactivity and deficits in PPI (summarized in **Table 3**).

Notably, vHC-hyperactivity has also been associated with *cognitive* deficits. *Sustained* attention [assessed on the 5-choice-serial-reaction time task, 5-CSRTT (Lustig et al., 2013)] is impaired by vHC disinhibition (McGarrity et al., 2017; Tan et al., 2018). Optogenetic activation of the ventral subiculum impairs spatial novelty-preference, a form of short-term memory that relies on the implicit detection of spatial novelty and its progressive habituation following continued exposure (Wolff et al., 2018). Also, cognitive flexibility and spatial working memory are impaired in a variety of models with elevated vHC and dopaminergic activity (**Table 4**). These results suggest, that aberrant ventral hippocampal activity may contribute not only to positive, but also to cognitive symptoms of schizophrenia [reviewed in (Bast et al., 2017)], even though it should be noted that most models listed in **Table 4** do not represent manipulations that are exclusively localized to the vHC.

**Table 4:** Rodent models with aberrant dopaminergic activity and salience attribution.

Model	DA	HC	NiHL	PPI	Vig/Attn	RevL	SetShift	SWM	SNP	References
Gria1^-/-^	↑	(↑)?	↑	↓	–	↓	–	↓	↓	(Wiedholz et al., 2007; Barkus et al., 2014; Bygrave et al., 2019)
CD2^-/-^	↑	↑	↑	–	↓*	↓	↓	↓	→	(Gilani et al., 2014; Grimm et al., 2018)
vSub↑	↑	↑	↑	↓	↓	–	–	(↓)**	↓	See text & **Table 3**
MAM	↑	↑	–	↓	–	↓	↓	↓	–	(Lodge and Grace, 2009; Gastambide et al., 2012)
Ketamine	↑	↑	↑	↓	–	↓	↓	↓	–	(Becker et al., 2003; Chatterjee et al., 2011; Chatterjee et al., 2012; Featherstone et al., 2012; Kittelberger et al., 2012; Schobel et al., 2013; Szlachta et al., 2017; Lee and Zhou, 2019)

↑ increase of indicated function; → unaltered function; ↓, decrease of indicated function; DA, mesolimbic dopaminergic activity; HC, hippocampal activity; NiHL, novelty-induced hyperlocomotion; PPI, pre-pulse inhibition; RevL, reversal learning; SetShift, set-shifting or rule-shifting task; Vig/Attn, sustained attention (vigilance) assessed in the 5-CSRTT; SWM, spatial working memory (maze-based); SNP, spatial novelty-preference (Y-maze); Ketamine, chronic or acute ketamine application; vSub↑, model involving electrical, chemical or optogenetic stimulation of the ventral subiculum. *CD2-KO mice show normal attentional performance under baseline but are somewhat stronger impaired when challenged (Grimm et al., 2018). **vHC disinhibition impairs 1-trial spatial memory in the water-maze (McGarrity et al., 2017).

It needs to be noted that similar impairments are also seen with hippocampal *lesions*, supporting the notion that not simply *reduced* but *intact* hippocampal processing is critical to set appropriate levels of salience attribution. For example, enhanced novelty-induced hyperlocomotion and impairment of spatial novelty-preference are caused by hippocampal lesions in adulthood (Sanderson et al., 2009). Also, neonatal vHC lesion enhances novelty-induced hyperactivity and decreases PPI in adulthood (Lipska and Weinberger, 2000; Placek et al., 2013; Cabungcal et al., 2014).

## An Additional Route for Controlling Dopamine: CA3→LS→VTA

**Figure 3:**
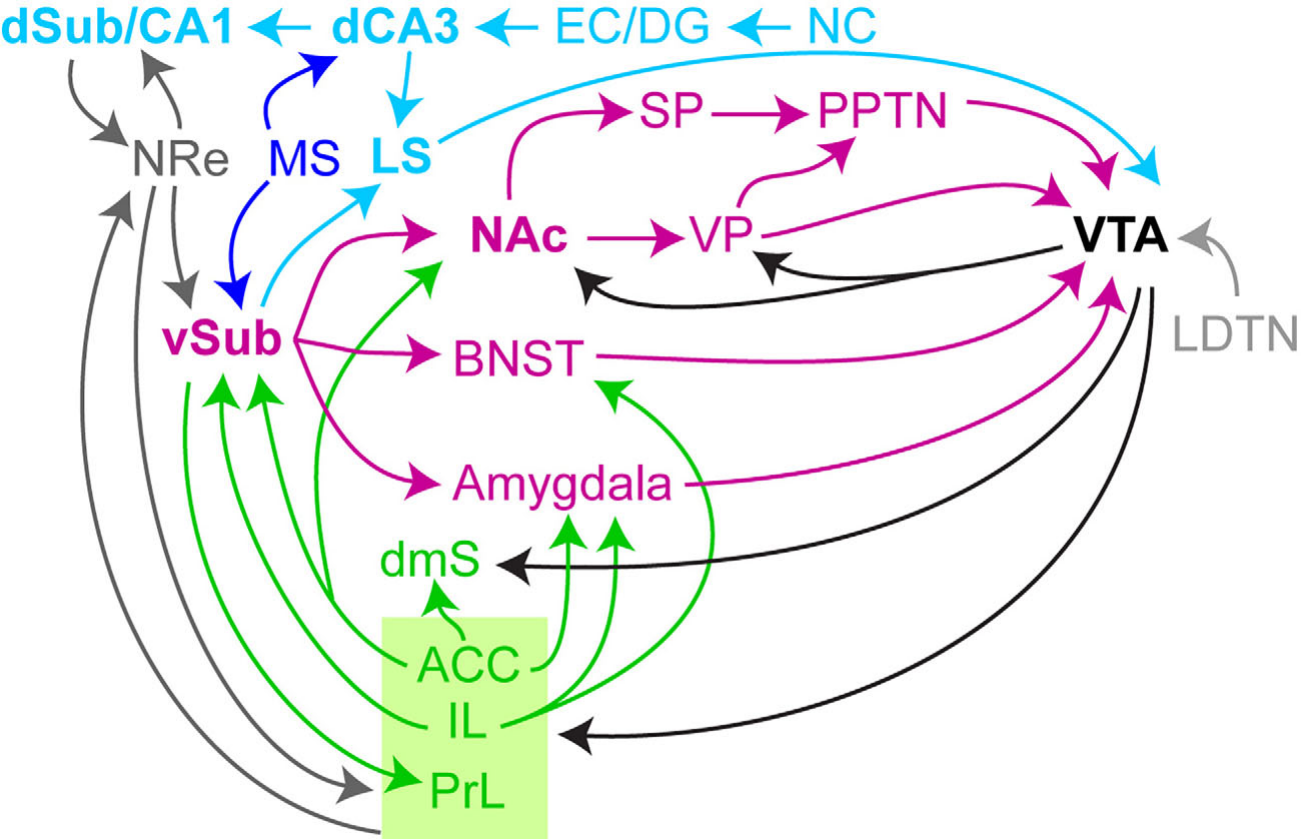
Dorsal hippocampal and septal projections that regulate dopamine neuron activity. Extension of **Figure 2**, additionally showing prominent connections from the dorsal CA3-subfield (*dCA3*), encoding contextual information from the neocortex (*NC*) transmitted through the entorhinal cortex (*EC*) and dentate gyrus (*DG*), to the lateral septum (*LS*). A further link between the dCA3 and the vSub circuit is mediated *via* dorsal CA1 (*dCA1*) and the nucleus reuniens (*NRe*).

While the evidence that the ventral subiculum is the crucial output channel for hippocampal control of the VTA is ample (**Tables 1**–**3**), it has been shown that the dorsal CA2/CA3 (dCA2/dCA3) region can also exert a significant excitatory influence on dopaminergic neurons of the VTA *via* the lateral septum (Luo et al., 2011) (**Figure 3**). Theta-frequency stimulation of dCA3 neurons increases the firing rate of VTA dopamine neurons (Luo et al., 2011). Direct optogenetic stimulation of medial-septum→dCA3 afferents at theta-frequency increases locomotor activity through the dCA3→lateral septum projection (Bender et al., 2015). These findings also link to the observation that many hippocampal molecular and cellular alterations in schizophrenia are localized in CA3 (Tamminga et al., 2010; Li et al., 2015).

This more direct route from the hippocampus to the VTA is closely inter-linked with the vSub→VTA circuit: they share a common *afference*, namely from the *medial septum* (Bannerman et al., 2004), and a common *efference*, namely to the *lateral septum* (LS). The LS has been shown to be the physiologically most prominent output of the ventral subiculum (Yang and Mogenson, 1984; Takata et al., 2015), and might therefore represent a “hub” for integration of dCA3 and vSub computational results before modulating the activity of neurons in the VTA.

Beyond the dCA3→LS projection, there are also at least two further convergence points through the canonical CA3→CA1→subiculum loop: firstly, the vHC is a prominent output region of the dHC (dorsal CA1/subiculum) (Takata et al., 2015), and secondly the dHC also projects reciprocally to the *nucleus reuniens* of the midline thalamus (Vertes, 2006; Griffin, 2015) (**Figure 3**). This projection is not only a relay to the prefrontal cortex (Vertes, 2006; Griffin, 2015; Hallock et al., 2016) but also to the vHC, since electrical stimulation of the nucleus reuniens increases VTA dopamine neuron activity *via* the vSub (Zimmerman and Grace, 2016). It remains to be determined, however, to what extent blunt elevation of the activity of the dorsal hippocampus may enhance mesolimbic dopamine signaling, as one study demonstrated that stimulation of the dHC—in contrast to the vHC—was not sufficient to evoke dopamine release in the NAc (Howland et al., 2004). Finally, there are also direct projections from the CA2/CA3-region of the *ventral* hippocampus to the VTA, whose physiological role remains to be investigated (Luo et al., 2011).

## A Hippocampal-Septal Circuit as a Comparator for Salience Detection

A key question concerns what specific role the hippocampus has in determining the dopaminergic salience signal. Several lines of evidence point to the possibility that the hippocampus contributes to the computation of the saliency of specific stimuli based on their novelty/familiarity. Early theories suggested that the hippocampus serves as a *comparator* which determines mismatches between expectation based on memory and current sensory experience, and gates attention and behavioral output accordingly (Gray and McNaughton, 1982). Different theories have been put forward, placing slightly different emphasis on the behavioral consequences of hippocampal processing [reviewed in chapter 9 of (Gray and McNaughton, 1982)]. For example, Douglas (Douglas, 1967) proposed that the hippocampus filters out *redundant* stimuli which are not predicting reward in order to prevent their influence on behavior. Vinogradova (2001) emphasized a hippocampal role for detecting novelty/familiarity during sensory processing. Two key empirical results supported her model: firstly, individual neurons in CA1 habituate their sensory-evoked responses with repeated presentation of the same stimulus [akin to the short-term habituation of CA1-BOLD in humans discussed above, (Holt et al., 2005)]; and secondly, this habituation was abolished and the response to familiar stimuli actually increased—(i.e. sensitized)—if CA1 was disconnected from CA3 (i.e. Schaffer collaterals were severed *in vivo*) (Vinogradova, 2001). In this view, the hippocampus detects matches or mismatches between expectations based on previous sensory experience (memories) and current sensory input, thereby computing a saliency or prediction error signal which would then be relayed to the VTA (Vinogradova, 2001). Particularly dorsal CA3-neurons encode context and its global changes (Alvernhe et al., 2008; Bannerman et al., 2014; Hainmueller and Bartos, 2018) and hence their output might be crucial for comparing the expected with the observed stimulus landscape (Luo et al., 2011).

Lesions of the whole hippocampus impair the detection of mismatches in sequences of sensory stimuli; these lesioned animals fail to show a renewed orienting response when the elements of two previously learned sequences of a specific sound followed by a specific visual cue are switched (Honey et al., 1998). Also, the proper adjustment of the orienting response according to novelty-related salience is impaired by these lesions (Marshall et al., 2004). Further, selective restoration of the expression of the GluA1-subunit of AMPA-receptors in the CA2/3-area of mice that globally lack them (Gria1^-/-^) could completely normalize their excessive novelty-induced hyperactivity and restore preference for spatial novelty (Bygrave et al., 2019). Notably, this GluA1^CA2/3^-rescue also normalized strongly elevated novelty-induced dHC theta-oscillations in this mouse model, which aligns with the findings described above that dCA3-theta activation strongly drives VTA dopamine neurons and associated hyperlocomotion (Luo et al., 2011; Bender et al., 2015). This finding also relates back to schizophrenia in general, because variants of the locus of the GluA1-encoding gene GRIA1 have been associated with increased risk for schizophrenia (Schizophrenia Working Group of the Psychiatric Genomics Consortium, 2014), and GluA1-mRNA expression is reduced in the CA3-region of SCZ patients (Eastwood et al., 1995).

## Targeting Hippocampal Hyperactivity Pharmacologically

The potentially central role of hippocampal hyperactivity in causing the hyperdopaminergic state in schizophrenia and resulting aberrant assignment of salience provides a rationale for attempts to reduce hippocampal excitability and synaptic glutamate release. Partially, this might be achievable by re-purposing existing drugs, for example by low doses of **levetiracetam** which reduces presynaptic transmitter release (Surges et al., 2008; Meehan et al., 2011), enhances GABAergic signaling (Wakita et al., 2014), and ameliorates age-related hippocampal hyperactivity (Haberman et al., 2017) (currently tested in clinical trials NCT03034356, NCT02647437).

Another strategy to potentially achieve this goal is the activation of presynaptically expressed inhibitory (G_i_-protein-coupled) metabotropic glutamate receptors type 2 and 3 (**mGluR2/3**). mGluR2/3 agonists can reduce abnormal ventral/anterior hippocampal hyperactivity produced by sub-chronic ketamine application in mice (Schobel et al., 2013). Prodrugs of an mGluR2/3 agonist or a selective mGluR2 agonist can both reduce elevated cortical activity induced by acute ketamine in healthy volunteers (Mehta et al., 2018). However, this effect is not specific to the hippocampus (Mehta et al., 2018) and also achievable with the ‘classic’ neuroleptic risperidone and the anti-epileptic lamotrigine (Doyle et al., 2013), and hence not necessarily indicative of superior therapeutic value of novel antipsychotics. Clinical trials with the prodrug of the mGluR2/3 agonist LY404039, *pomaglumetad methionil* (LY2140023), have not yielded significant improvement of symptoms of schizophrenia, however (Adams et al., 2013; Stauffer et al., 2013; Adams et al., 2014; Downing et al., 2014). Nevertheless, a later re-analysis of these data showed that patients at an early stage of the disease did actually benefit from the treatment (Kinon et al., 2015), which is in line with the notion that drugs targeting hippocampal hyperactivity need to be applied early in the disease process to stop the maladaptive learning processes driven by aberrant salience (Kapur, 2003) and the ensuing spreading, irreversible hippocampal atrophy (Schobel et al., 2013; Moghaddam, 2013; Ho et al., 2017). The ability of the mGluR2/3 agonist pomaglumetad to reduce hippocampal (CA1) activity (CBV) in prodromal patients will soon be evaluated (NCT03321617). Furthermore, more studies are required in animals to understand the nature of the relationship between mGluR2/3 agonists and dopamine levels, given the important role that these receptors may play at the interface between arousal and cognition (Lyon et al., 2011).

Another existing drug that has three independent mechanisms for reducing elevated glutamatergic excitation in cortical circuits—and is easily repurposed—is **N-acetyl-cysteine** (NAC). Firstly, NAC targets the cysteine-glutamate antiporter thereby increasing glutamate import into glia cells (Baker et al., 2008; Durieux et al., 2015; McQueen et al., 2018). Secondly, NAC leads to the release of extra-synaptic glutamate thereby preferentially activating mGluR2/3s (Conn and Pin, 1997; Zavodnick and Ali, 2014). Thirdly, NAC is a precursor of gluthathione (GSH) and therefore has anti-oxidant effects which have been shown to protect parvalbumin-positive inhibitory interneurons (PV-INs) and their extracellular environment (peri-neuronal nets) from oxidative stress; thereby NAC may prevent a potential disinhibition of cortical circuits which is expected to result from the hypofunction of these PV-INs (Lisman et al., 2008; das Neves Duarte et al., 2012; Cabungcal et al., 2014). Strengthening PV-IN function—whether through anti-oxidant effectors like NAC (Cabungcal et al., 2013) or inhibitors of the matrix-metallo-protease 9 (Dwir et al., 2019) or other mechanisms yet to be discovered—may be beneficial due to a reduction of hippocampal activity (Lisman et al., 2008) or due to improvement of attention-related gamma oscillations, for which these neurons are critical (Bartos et al., 2007; Cardin et al., 2009; Sohal et al., 2009; Cho et al., 2015). NAC improves symptoms across all three domains, including attention, short-term and working memory in humans (Lavoie et al., 2008; Sepehrmanesh et al., 2018), and its efficacy is supported by recent meta-analyses (Yolland et al., 2019; Çakici et al., 2019; Firth et al., 2019). This aligns with the NAC-induced rescues of deficits in the rodent neonatal ventral hippocampal lesion model (Cabungcal et al., 2014) and the phencyclidine model of schizophrenia (Baker et al., 2008). NAC has also been shown to reduce head-twitches induced by the 5-HT2A/C agonist DOI, which serves as a serotonergic model of hallucinations in psychosis—and moreover, it does so through an mGluR2-dependent mechanism (Lee et al., 2014).

A further relevant compound is the glutamate-release inhibitor **riluzole** (de Boer et al., 2019) [although see (da Silva et al., 2003)]. Riluzole was effective in decreasing glutamate/glutamine (Glx) levels in anterior cingulate cortex (hippocampus was not reported) in treatment-resistant patients with schizophrenia, in whom Glx-levels also correlated with negative and cognitive symptoms (Pillinger et al., 2019). Furthermore, riluzole-treatment was effective in reducing negative symptoms in patients with schizophrenia in a small-scale clinical trial (Farokhnia et al., 2014). Although the clinical experience with riluzole in schizophrenia is very limited, further studies are warranted.

Further drug targets may be discovered through the strategy laid out in **Figure 1**—if applied to the circuits described in this review. The large number of brain regions involved in regulating the dopaminergic midbrain—including, but not limited to the hippocampus—and the rich plethora of cell-types within those structures provides ample candidate cell populations whose modulation may ameliorate maladaptive dopamine release. Such cellular targets can be translated into molecular targets by cell-type specific gene expression analysis to identify selectively expressed genes that modulate their neural activity.

## Limitations and Challenges of Harnessing the Hippocampus→NAc→VTA Circuit for Anti-Psychotic Action

While numerous major brain regions have been implicated in schizophrenia—including the neocortex, thalamus, and cerebellum which were not particularly highlighted here—singling out the hippocampus as a potential therapeutic target seems justified by the robustness of its aberrations in schizophrenia and its validated role in controlling dopaminergic signaling. Nevertheless, this framework has its caveats, limitations, and remaining questions.

Can amelioration of hippocampal hyperactivity correct aberrations in *other* brain regions, especially if conducted, not as an early intervention, but in established schizophrenia? Is the dorsal/posterior or the ventral/anterior hippocampus to be targeted (or both)? Patient data suggest that hyperactivity of the *anterior* hippocampus is the primary aberration (Schobel et al., 2013). This is supported by the large body of evidence demonstrating the control of dopaminergic activity by the vHC and vSub in rodents, and the potential therapeutic effects achieved by local interventions in the vHC [**Tables 1** and **3**, (Gilani et al., 2014)]. However, salience-like signals in rodents have mostly been recorded in the dorsal hippocampus which receives predominantly sensory information, and more successful rescue approaches which reduce hippocampal hyperactivity in genetic mouse models of schizophrenia have been conducted in the dorsal subregion (Marissal et al., 2018; Aitta-aho et al., 2019; Mukherjee et al., 2019) than in the vHC (Gilani et al., 2014). Therefore, further elucidation of the different functional roles of the dorsal and ventral subregions in schizophrenia-related deficits remains a pressing need. This regards in particular their respective roles in processing the sensory and emotional salience of stimuli, and the mechanisms of their interaction (Bannerman et al., 2004; Bannerman et al., 2014).

Additionally, the vast complexity of the brain-wide circuit controlling dopamine (**Figure 3**) may entail the risk that interventions targeted at one region are outweighed by pathologies in another one, and even that among schizophrenia patients there is enormous heterogeneity, with subpopulations in whom aberrant salience may be unrelated to the hippocampus and which would hence remain unresponsive to such a therapy. This key complication may explain why some drugs, that are very promising, not only pre-clinically but also in small-scale Phase II trials, may fail in larger Phase III trials (Kinon et al., 2015). Without patient-stratification according to appropriate—yet to be validated—physiological, behavioral, or genetic biomarkers it might prove very difficult to bring any compound that is based on this mechanism to the clinic. Our current approach to clinical trials resembles a garage that tries to repair all cars by replacing the same part of the motor, irrespective of why each individual vehicle does not actually drive anymore. Not only the large number of schizophrenia risk genes (Schizophrenia Working Group of the Psychiatric Genomics Consortium, 2014), but also the variety of neurotransmitter systems and brain regions involved in salience attribution (**Figure 3** and beyond)—in addition to the heterogeneity of cell types within these regions (not covered in this review)—highlights the vast number of potential “break points” of the salience assignment system in the brain, and underscores the need for more personalized interventions.

A further caveat is that dopamine may not necessarily be the only final common pathway of salience attribution. D2-antagonism is not sufficient to fully normalize hyperlocomotion induced by electrical (Taepavarapruk et al., 2000) or optogenetic (Wolff et al., 2018) stimulation, nor novelty-induced hyperlocomotion in cyclin D2-knockout mice which show hippocampal hyperactivity (Gilani et al., 2014; Grimm et al., 2018). Even the induction of hyperlocomotion and striatal overactivation produced by systemic blockade of NMDA-receptors (MK-801 or PCP) does not require dopaminergic transmission from VTA/SNc—as shown in mice that lack tyrosine hydroxylase in dopaminergic neurons—and can be rescued by the mGluR2/3-agonist LY379268 (Chartoff et al., 2005). Finally, patients with treatment-resistant schizophrenia—in contrast to treatment-responsive patients—do not show increased dopamine synthesis capacity in any subdivision of the striatum (Demjaha et al., 2012). These results do not necessarily invalidate the usefulness of interventions that aim to reduce hippocampal excitability. For example, in the cyclin D2-knockout model, LY379268, in contrast to anti-dopaminergic treatment, could fully normalize hyperlocomotion (Grimm et al., 2018).

These observations point to the possibility that multiple other circuits may also regulate salience attribution (**Figure 3**), such as *via* other neuromodulators. For example, the noradrenergic locus coeruleus (LC) plays a role in modulating attention in prefrontal circuits (Aston-Jones and Cohen, 2005; Arnsten, 2011) and in directly controlling the activity of a subpopulation of vSub neurons (Lipski and Grace, 2013a) in response to salient stimuli (Lipski and Grace, 2013b). Its activation has been associated with salience processing and with the change of activity in salience-related brain structures (Yu and Dayan, 2005; Zerbi et al., 2019), including with the induction of long-term plasticity of CA3→CA1 synapses (Lemon et al., 2009). Notably the LC is also a major source of dopamine for all subfields of the dHC (while only CA2 receives dopamine from the VTA) (Kempadoo et al., 2016; Takeuchi et al., 2016). Likewise, serotonin has long been implicated in salience attribution (especially aversive salience) and psychosis. 5-HT (serotonin) receptors are targeted by multiple antipsychotics, and the specific 5-HT2A antagonist pimavanserin has recently been approved for the treatment of psychosis in parkinsonian patients (Bozymski et al., 2017; Kianirad and Simuni, 2017; Sahli and Tarazi, 2018).

Aside from the involvement of neuromodulatory systems other than dopamine, other *brain structures* than those critically engaged in controlling the VTA have been implicated in salience regulation. Most prominently, the anterior cingulate cortex (ACC) encodes reward predictions and reward prediction errors (Bissonette and Roesch, 2016), receives input not only from the VTA (Decot et al., 2017) but also the LC (Fillinger et al., 2017), and projects to the associative and—to a lesser extent—the ventral striatum (Fillinger et al., 2018) (**Figure 2**). The latter (ACC→NAc) connection is functionally reduced in first-episode schizophrenia (Lin et al., 2018). The ACC also projects to further key structures of the extended vSub/VTA circuit, such as the amygdala and the vHC (**Figure 2**)—these connections are crucial for attribution of the appropriate levels of significance during associative learning (Bian et al., 2019; Ortiz et al., 2019).

## Conclusions

The elucidation of neuronal cell-types that are key regulators of salience attribution and subsequent identification of selectively expressed genes within them, promises to lead to new molecular targets for treating psychosis. The specific probing of dopamine-regulating and salience-assigning circuits with remote cell-type specific manipulations within them provide a unique opportunity for the drug discovery process.

However, something that needs to be considered carefully when contemplating such therapeutic approaches is that salience-attribution needs to be not simply higher or lower but *appropriate*, in order to prevent maladaptive learning. Both inappropriately high and inappropriately low salience will impact on the accuracy of learning processes. For example, the discussed studies which reported physiological patterns of salience attribution, illustrated this enormous complication by demonstrating that such patterns are entirely altered in patients with schizophrenia. Therefore, strategies for early interventions that can prevent maladaptive learning processes induced by aberrant salience and halt degeneration of the hippocampus (Schobel et al., 2013; McHugo et al., 2018) deserve particular priority (Sommer et al., 2016).

## Author Contributions

DK and DB drafted the manuscript. All authors contributed to the article and approved the submitted version.

## Funding

Related research in the laboratories of the authors was or is supported by the Wellcome Trust (DB, DK, AB), the Roche Postdoctoral Fellowship Programme (AW, DK, grant RPF 247), the Brain and Behaviour Research Foundation (NARSAD, DK, grant 22616), the Junior-Professorship programme of Baden-Württemberg (DK), the Else-Kröner-Fresenius/German-Scholars-Organization Programme for excellent medical scientists from abroad (DK, grant GSO/EKFS 12), the DFG (DK, grant KA 4594/2-1) and the Medical Research Council UK (DB, grant MR/N004396/1).

## Conflict of Interest

The authors declare that the research was conducted in the absence of any commercial or financial relationships that could be construed as a potential conflict of interest.
